# Allergic reaction related to ramipril use: a case report

**DOI:** 10.1186/1758-5996-2-4

**Published:** 2010-01-20

**Authors:** Renata C Alencar, Roberta A Cobas, Marília B Gomes

**Affiliations:** 1 Department of Endocrinology and Metabology, Hospital Universitário Pedro Ernesto, Universidade Estadual do Rio de Janeiro (UERJ), Rio de Janeiro, RJ, Brazil

## Abstract

**Background:**

Angiotensin-converting enzyme (ACE) inhibitors are widely prescribed for patients with diabetes as a nephroprotector drug or to treat hypertension. Generally they are safe for clinical practice, but the relationship between these drugs and angioedema is known. The exact mechanism for ACE inhibitors-induced angioedema is not clear and it is still a matter of discussion.

**Case Report:**

We reported a case of a 23-year-old black female with an 11 year history of type 1 diabetes, regularly monitored in the department of diabetes, in use of 0,98 UI/kg/day of human insulin, which presented an allergic reaction 24 h after ramipril use. The drug had been prescribed to treat diabetic nephropathy. There was no previous history of drug induced or alimentary allergy. The patient was instructed to discontinue the use of ramipril and oral antihistaminic drug and topical corticosteroid were prescribed. Skin biopsies were performed and confirmed the clinical hypothesis of pharmacodermy. The evaluation of ACE polymorphism identified *DD *genotype. Six months after the withdrawal of ramipril the patient was prescribed the angiotensin-II receptor blocker (ARB) losartan as nephroprotector. She remained well without adverse reactions.

**Conclusions:**

ACE inhibitors-induced angioedema is uncommon and the clinical presentation is variable with lips, tongue, oropharinge, and larynge as the most common locations. The presence of angioedema during treatment requires the immediate cessation of treatment due to the risk of possible severe complications. The case reported presented moderate symptoms, with the development of early onset edema in uncommon regions. ACE *DD *genotype had been associated with angioedema-ACE inhibitors induced. In patients who have experienced ACE inhibitor-related angioedema, ARB should be used cautiously used. However in the case of our patient, the prescription of losartan as nefroprotector did not result in any recurrent adverse effect.

## Background

Angiotensin-converting enzyme (ACE) inhibitors are widely prescribed for patients with diabetes as a nefroprotector drug or to treat hypertension. Generally, they are safe for clinical practice, but the relationship between these drugs and angioedema is known.

We report a case of a young female diagnosed with type 1 diabetes that developed angioedema and drug reaction after administration of ramipril.

## Case Report

A 23-year-old black female with an 11 year history of type 1 diabetes, was admitted to the hospital 5 days after the appearance of pruriginous erythemato-vesiculo-papulous eruptive lesions in abdomen with later generalization. Some lesions presented local bleeding and she noted swelling of the face and ears. There was no history of drug induced or alimentary allergy. She was using 0,98 UI/Kg/day of human insulin and had started the use of the ACE ramipril 5 mg 24 hours before the symptoms occurred (prescribed to treat diabetic nephropathy). The patient presented normal renal function, weight and blood pressure levels. The evaluation of ACE polymorphism identified DD genotype.

On admission she was afebrile, normotensive and had no respiratory distress. On examination there were erythemato-papular lesions in abdomen and in posterior region of the thighs, some confluents and with vesicles. She presented periorbital swelling and erythematosus-swelling lesions in ears.

A clinical angioedema and an allergic reaction to ramipril were suspected. The patient was instructed to discontinue the use of ramipril, and oral antihistaminic drug and topical mometasone furoate were prescribed. Skin biopsies were performed in abdominal lesions.

The lesion regression was gradual and after 3 months there was residual hypercromy in previously injured site.

Later the histopathological findings confirmed the clinical hypothesis of pharmacodermy related to ramipril that manifested as erythemato-papular lesions and angioedema.

Six months after the withdrawal of ramipril the patient was prescribed the angiotensin-II receptor blocker losartan 25 mg OP as nephroprotector. She remained well without adverse reactions.

## Discussion

Angiotensin converting enzyme (ACE) inhibitors have been widely used in the treatment of cardiovascular and renal diseases. They are known to cause dry cough, hypotension, hyperkalemia and angioedema as adverse effects [1,2].

Diabetic nephropathy is a common cause of kidney failure and it is important to treat correctly. Diet and drugs that block the renin-angiotensin-aldosterone system are prescribed in early stage [3]. On advanced stage, dyalisis and renal transplantation are treatment options. Recent publication showed that type 1 diabetic patients show higher survival rates after transplant in comparison to the dialysis therapy [4].

The case reports a young, black female diagnosed with type 1 diabetes who developed angioedema and pruriginous reaction 24 hours after starting ramipril use to treat diabetic nephropathy.

ACE inhibitors-induced angioedema is uncommon. The incidence is very low (0,1 - 0,2%), but it is quite underestimated because of poorly recognized presentation, especially because of its late onset [5]. It most commonly affects African Americans, women [6,7] and smokers [7].

The onset of angioedema is variable; it can occur within the first 24 hours of ACE inhibitors use, however weeks, months and years have also been described. These variable temporal relationships between drug administration and adverse effects can contribute to a failure in recognizing the association and discontinuation of ACE inhibitors [7].

The ACE inhibitors can induce urticariform reactions, bullous lesions and phototoxic reactions, in particular captopril which contains a thiol group [8] as with angioedema, usually without associated urticaria [8]. Some authors reviewing charts of patients presenting angioedema to an emergency department have observed that pruritus was noted only in patients with angioedema not related to the use of ACE inhibitors [9]. The most common locations for angioedema-ACE inhibitors induced are lips, tongue, oropharinge, and larynge. In the last case the patient could eventually need intubation [10]. Others drugs commonly prescribed for diabetic patients may also cause cutaneous reaction (table 1).

**Table 1:** Drugs-related cutaneous reaction (Drugs widely prescribed for diabetic patients)

ACE	Urticariform reactions (8;23); angioedema (8;23); bullous eruptions*(8;23); phototoxic reaction* (8;23); morbiliform eruption [24]
ARB	Urticariform reactions; angioedema [22]

Acetylsalicylic Acid	Urticaria [23]; angioedema [23]; Erythema polymorphous [24]

Thiazide	Photosensitivity [23]; morbiliform eruption [24]

Insulin	Papular Erythema [25]; urticaria [25]

Beta Blocker	Drug induced lupus [24]; lichenoid eruption**[24]

*Only Captopril

**Only Propranolol

The case reported presented moderate symptoms, with the development of edema in uncommon regions, such as the external ear and eyelids, besides the presence of pruriginous lesions in abdomen and legs. In a New Zealand study, the authors reviewed reports of angioedema and urticaria associated with ACE inhibitors. Of a total of 116 reports, 90% developed angioedema or urticaria alone and 10% presented both manifestations, without sex difference [11].

ACE inhibitors can cause non-immune, idiopatic angioedematous and urticariform reactions, mediated by complement system and others plasmatic systems. However, the exact mechanism for ACE inhibitors-induced angioedema is not clear and it is still a matter of discussion. Cyclo-oxygenase pathway activation, with mastocyte degranulation is described [12]. Some authors attribute the ACE-inhibitor-related angioedema to the increased levels of bradykinin [6,13,14] and substance P [15]. The increased levels of bradykinin are not seen with angiotensin II receptor blocker (ARB). However, angioedema associated with ARB has also been observed, although it is less common [16].

A study group proposed that ACE gene polymorphism and some enzyme deficiencies could be involved in ACE inhibitor-induced angioedema [5]. The amount of immunoreactive ACE [17,18] and catalytic activity [19] are related to I/D genetic polymorphism. In Chinese patients there is evidence that genetic polymorphism is associated with a structural alteration in ACE [19]. However, recent data showed no relationship between ACE gene polymorphism and the occurrence of angioedema with ACE and ARB [20]. One study analyzed the metabolism of endogenous bradikinin (B2 receptor agonist) and its active metabolite, des-Arg9-Bradikinin (B1 receptor agonist) in the presence of an ACE-inhibitor during *in vitro *contact activation of plasma from hypertensive patients who presented angioedema and these kinetic parameters were compared with those measured in a control group (without angioedema) of hypertensive patients. They evidenced that the slope of bradikinin degradation was lower in patients with angioedema, such as des-Arg9-Bradikinin accumulation of the B1 agonist in these patients, when compared to controls, suggesting that angioedema pathogenetic mechanism lies in the catabolic site of kinin metabolism [21].

The presence of angioedema during treatment requires the immediate cessation of treatment due to the risk of possible severe complications [8]. If the drug is not discontinued after the initial episode of angioedema, a recurrence of episodes might occur [16]. In patients who have experienced ACE inhibitor-related angioedema, angiotensin receptor blockers should be used cautiously [22]. Some consider its use contra-indicated [8], but only a small percentage of patients with ACE inhibitor-related angioedema continue with this symptom when switched to an angiotensin II receptor blocker. In the case of our patient, the prescription of Losartan as nefroprotector did not result in any recurrent adverse effect.

## Conclusion

We report a case of a black female diagnosed with type 1 diabetes that developed a clinical manifestation of angioedema and drug reaction of early onset related to the use of ramipril. The patient presented improvement of the episode after substitution of the ACE inhibitor and clinical treatment with oral anti-histamine and topic corticosteroids and no recurrent symptoms after use of losartan.

## Consent

Written informed consent was obtained from the patient for publication of this case report. A copy of the written consent is available for review by the Edito-in-Chief of this journal.

## Competing interests

The authors declare that they have no competing interests.

## Authors' contributions

RCA and RAC participated in acquisition of data and drafting the manuscript.

MBG participated in revising critically the manuscript and giving the final approval of the version to be published.

All authors read and approved the final manuscript.
